# Is histone acetylation the most important physiological function for CBP and p300?

**DOI:** 10.18632/aging.100453

**Published:** 2012-04-16

**Authors:** David C. Bedford, Paul K. Brindle

**Affiliations:** Department of Biochemistry, St. Jude Children's Research Hospital, Memphis, TN 38105, USA

**Keywords:** CBP, p300, CREBBP, EP300, histones, acetylation, chromatin, transcription, coactivators, epigenetic, cancer, CREB, cyclic AMP

## Abstract

Protein lysine acetyltransferases (HATs or PATs) acetylate histones and other proteins, and are principally modeled as transcriptional coactivators. CREB binding protein (CBP, CREBBP) and its paralog p300 (EP300) constitute the KAT3 family of HATs in mammals, which has mostly unique sequence identity compared to other HAT families. Although studies in yeast show that many histone mutations cause modest or specific phenotypes, similar studies are impractical in mammals and it remains uncertain if histone acetylation is the primary physiological function for CBP/p300. Nonetheless, CBP and p300 mutations in humans and mice show that these coactivators have important roles in development, physiology, and disease, possibly because CBP and p300 act as network “hubs” with more than 400 described protein interaction partners. Analysis of CBP and p300 mutant mouse fibroblasts reveals CBP/p300 are together chiefly responsible for the global acetylation of histone H3 residues K18 and K27, and contribute to other locus-specific histone acetylation events. CBP/p300 can also be important for transcription, but the recruitment of CBP/p300 and their associated histone acetylation marks do not absolutely correlate with a requirement for gene activation. Rather, it appears that target gene context (e.g. DNA sequence) influences the extent to which CBP and p300 are necessary for transcription.

## Histone modifications often correlate with the state of gene expression

The canonical nucleosome is a fundamental unit of chromatin and consists of a protein octamer of two molecules each of histone H2A, H2B, H3 and H4, wrapped by 147 bp of genomic DNA. In this context, numerous histone post-translational modifications (PTMs) have been described that include acetylation, phosphorylation, methylation, ubiquitylation and SUMOylation [1]. Histone PTMs often correlate with the activity of the gene (or of a regulatory element such as an enhancer) that is in their proximity, suggesting a causal relationship. Relevant to the role of CBP/p300 as HATs, hyperacetylation of histone N-terminal tail lysines correlates strongly with active transcription [2]. Similarly, the recruitment of protein acetyltransferases positively correlates with histone hyperacetylation at active genes as shown by genome-wide chromatin immunoprecipitation (ChIP-Seq) studies of CBP and p300 in human T cells [3].

## Practical difficulties hamper the *in vivo* analysis of histone mutations in mammals

The function of histone PTMs in mammals remains uncertain because the multiple genes encoding each canonical histone, renders *in vivo* mutational analysis unfeasible in most instances. Exceptions to this limitation occur when analyzing histone variants that have few gene copies, or when assessing putative gain-of function histone mutations, such as those identified in pediatric glioblastoma and glioma (e.g. H3.3 K27M) [4,5]. For instance, knockout of the H2A variant H2A.Z reveals that it is required for early mouse development [6], whereas loss of another variant, macroH2A1, has subtle effects on mouse physiology and gene expression [7,8]. The difficulty of testing histone point mutations in mammals has therefore contributed to the uncertainty of whether canonical histone PTMs are correlative with gene expression or causal.

## Yeast histone mutants suggest subtle or specific roles for many histone PTMs

In baker's yeast, however, histone mutations can be easily made and numerous studies show that histone PTMs might not be as essential as their correlative behavior with transcription would suggest [9-14]. For instance, a systematic analysis of 486 different histone H3 and H4 mutations in yeast (where every residue was mutated at least one way) showed that only 11 of 79 N-terminal tail deletions resulted in lethality, a phenotype that also depends to some extent on strain background [9]. (In the filamentous fungus *Neurospora crassa*, however, certain histone H3 mutants are not viable, such as K4L, K9L, K14R and K27L [15]). Phenotype and gene expression changes are often surprisingly moderate or specific in yeast that harbor point mutations in the N-terminal tails of histones H2A, H2B, H3 and H4 [9,16-19]. For example, simultaneously mutating eight modifiable H3 lysines (K4, K9, K14, K18, K23, K27, K36, and K79) to glycine has no effect on the growth rate of yeast in synthetic complete glucose medium [20]. Such results using yeast suggest that in mammals, histone PTMs might not always be essential for nearby gene expression and that gene-proximal recruitment of a histone-modifying enzyme might sometimes be correlative with transcription rather than causative.

## HATs and histone acetylation

Controlled by the opposing actions of acetyltransferases (HATs) and deacetylases (HDACs), histone lysine acetylation is modeled to facilitate transcription by acting as a mark that affects the modification of other nearby residues, enhancing cofactor recruitment (e.g. via bromodomain containing proteins that bind acetyl-lysine), and by relaxing DNA/histone interactions by neutralizing lysine sidechain positive charge. There are four main multi-gene families of mammalian HATs based on sequence similarity: GCN5 and PCAF (encoded by *Gcn5l2* and *Pcaf* in mice), the MYST family (*Htatip*,*Myst1*, *Myst2*, *Myst3* and *Myst4*), the nuclear (or steroid) receptor coactivator family (*Ncoa1*, *Ncoa2*, *Ncoa3*, which may include *Clock*), and the CBP and p300 family (*Crebbp* and*Ep300*) [21]. While HATs within each family tend to share a high degree of sequence similarity, HAT enzymatic domain sequences are surprisingly dissimilar between the four main families [22,23]. Such divergence between HAT families suggests that they evolved for functions distinct from acetylating histone lysine residues. Consistent with this idea, HATs are also called protein acetyltransferases (PATs), and are known to modify many other nuclear, cytoplasmic and mitochondrial proteins [24]. Consequently, HATs have been reclassified as KATs (lysine or K-acetyltransferases) to more accurately reflect their varied protein substrates [25].

## CBP and p300 constitute the KAT3 family of HATs

**Figure 1:**
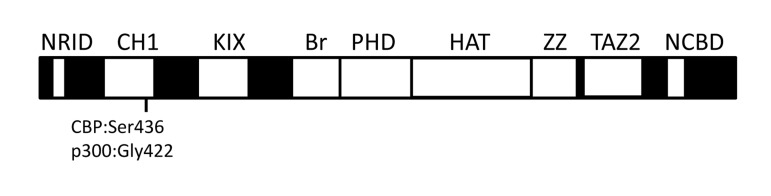
The relative location of conserved domains in CBP and p300. NRID (nuclear receptor interaction domain), CH1 (cysteine/histidine-rich region 1, also known as transcriptional-adaptor zinc-finger domain 1 or TAZ1), KIX (kinase inducible domain of CREB interacting domain), Bromodomain (Br), PHD (plant homeodomain), HAT (histone acetyltransferase domain), ZZ (ZZ-type zinc finger domain), TAZ2 (transcriptional-adaptor zinc-finger domain 2; ZZ and TAZ2 together are sometimes referred to as CH3 or cysteine/histidine-rich region 3), and NCBD [nuclear coactivator binding domain or IRF3-binding domain (IBiD)] [26,27,86]. Regions in black indicate the largely nonconserved and unstructured sequences between the conserved domains (white boxes). Locations of Ser436 (Ser437 in humans) in the mouse CBP CH1 domain and Gly422 (Gly421 in humans) in the corresponding position of p300 are indicated. Not drawn to scale.

CBP (CREBBP or CREB binding protein) and p300 (EP300 or E1A binding protein p300) interact physically or functionally with over 400 different proteins and together form the two-member KAT3 family of histone acetyltransferases [21] (internet search “CBP-p300 interactome” for an updated list with references). Their ability to interact with so many proteins occurs via several conserved protein binding domains [i.e., NRID, CH1 (TAZ1), KIX, Bromodomain, PHD, HAT, ZZ, TAZ2 and the NCBD (IBiD)] (Figure 1) [26,27]. Most of these domains are unique to CBP and p300, at least at the level of primary sequence. The large repertoire of interacting partners makes CBP/p300 among the most heavily connected nodes in the known mammalian protein-protein interactome [21].

## CBP and p300 are essential for normal human development

Analysis of global transcription networks in model organisms indicates that proteins that function as nodes or “hubs” are often encoded by essential genes [28]. Consistent with designating CBP and p300 as “hubs”, they are each required for normal development and are implicated in human disease. The archetype human disorder associated with a CBP or p300 mutation is Rubinstein-Taybi syndrome (RTS), a congenital developmental disorder characterized by growth impairment, mental retardation, and distinctive facial and skeletal anomalies [29]. The identification of heterozygous mutations of CBP provided the first evidence that RTS is caused by a deficiency in CBP protein function (i.e., haploinsufficiency) [30]. A subsequent screen of 92 RTS patients revealed that 36 had mutations in CBP and three had mutations in p300 [31]. In fact, several RTS-associated mutations have been found to affect CBP enzymatic activity, indicating that reduced HAT function could underlie the syndrome [32,33]. However, at present the detailed molecular mechanisms that cause RTS are unknown.

## Nullizygous mutations of either CBP or p300 result in early embryonic lethality in mice

*CBP^−/−^*, *p300^−/−^* and *CBP^+/−^*;*p300^+/−^* mice all die during embryogenesis, with the compound heterozygous phenotype indicating that the combined amount of the two proteins is limiting [34,35]. Interestingly, *CBP^+/−^* mice exhibit characteristics of RTS, including growth retardation and craniofacial anomalies [36,37], implying that some of the developmental functions of CBP are conserved between mice and man [36-40]. In contrast, *p300^+/−^* mice are slightly smaller and less thrifty than wild-type littermate controls but are otherwise grossly normal [35].

The early embryonic lethality observed in CBP and p300 knockout mice complicated efforts to understand the role of CBP and p300 in adult cell lineages. Subsequently, the creation of conditional Cre/LoxP knockout alleles (*CBP^flox^* and *p300^flox^*) has helped overcome this problem. Studies using conditional knockouts indicate that CBP and p300 individually can have distinct roles in defined cell lineages, although the loss of both genes is highly detrimental to cell proliferation [41-44].

## CBP and p300 mutations in cancer

Several recent studies have identified somatic mutations that alter CBP and p300 activity in a significant fraction of patients with B-cell non-Hodgkin's lymphoma [45-47], relapsed acute lymphoblastic leukemia [48], and transitional cell carcinoma of the bladder [49]. These findings are consistent with the observation that RTS patients have an increased susceptibility for tumor development [50,51].

## Are CBP and p300 counter-regulators of protein deacetylases in the control of aging and metabolism?

Activation of sirtuin protein deacetylases is postulated to help mediate the effects of caloric restriction and reduced insulin signalling that promote longevity [52]. Presumably, HATs are the counter-regulators of sirtuin activity in this physiologically context through their ability to acetylate proteins that are targeted by HDACs. Although the role of HATs in the aging process is less well understood than it is for sirtuins, it is known that depleting CBP in *C. elegans* blocks the lifespan extension induced by dietary restriction and hypothalamic expression of CBP is reportedly reduced in aging mice [53]. Such studies suggest that CBP HAT activity is a determinant of healthy aging. In keeping with this notion, *CBP^+/−^* mice are also lean and insulin-sensitized [54] and growing evidence places CBP/p300 in signalling pathways capable of promoting energy homeostasis [55,56]. For example, p300 acetylates the energy-state-sensor AMP kinase to inhibit its activity and promote lipid storage; AMPK acetylation is counter-regulated by the deacetylase HDAC1, which promotes lipid breakdown [57]. Accordingly, CBP and p300 are receiving growing attention as potential therapeutic targets for the treatment of metabolic diseases and other age-related pathologies.

## CBP and p300 are important for whole animal energy homeostasis

During fasting, glucagon is secreted from the pancreas and promotes hepatic glucose production (HGP) by increasing liver intracellular cAMP and gluconeogenic gene expression [58]. Hepatic gluconeogenic gene transcription is stimulated via recruitment of HAT (CBP/p300) and non-HAT (CRTC, CREB Regulated Transcription Coactivator) coactivators to CREB that is bound to target gene promoters [59,60]. However, fasting blood glucose levels and hepatic gluconeogenic gene expression are unaltered in *CBP^KIX/KIX^*mice, which carry point mutations that block the interaction between the CBP KIX domain and CREB [61]. This indicates that the main interaction between CBP and CREB is not limiting for hepatic gluconeogenesis [60]. Conversely, mice with a serine-to-alanine mutation in the CH1 domain of CBP (Ser436Ala) display increased HGP, and are resistant to the hypoglycemia-inducing effects of insulin and metformin [55,62]. This implicates the CBP CH1 domain in glucose homeostasis, suggesting that Ser436 phosphorylation can negatively regulate the interaction between CBP and CREB. Moreover, as p300 lacks a serine at the equivalent position in its CH1 domain (Figure 1), it was suggested that CBP has unique insulin- and metformin-responsive properties and is limiting for liver gluconeogenesis [55,62]. However, it was recently demonstrated that conditional knockout of CBP in the liver does not decrease fasting blood glucose or gluconeogenic gene expression in mice [56]. Similarly, fasting blood glucose levels, liver gluconeogenic gene expression and metformin responsiveness were all unaffected in mice homozygous for an in-frame deletion mutation in the CH1 domain of CBP (*CBP^Δ^^CH1/^^Δ^^CH1^*) or p300 (*p300^Δ^^CH1/^^Δ^^CH1^*) [56]. In fact, ΔCH1 mutant mice are lean and insulin-sensitized, suggesting that an intact CH1 domain structure is necessary for normal energy storage, but not the glucose lowering actions of insulin and metformin. Collectively, these findings are consistent with the notion that CBP is not limiting for hepatic gluconeogenesis, and that other coactivators can compensate for loss of CBP function at CREB target genes (e.g. p300, CRTC2).

## Insights from CBP and p300 hypomorphic mutations created in mice

**Figure 2:**
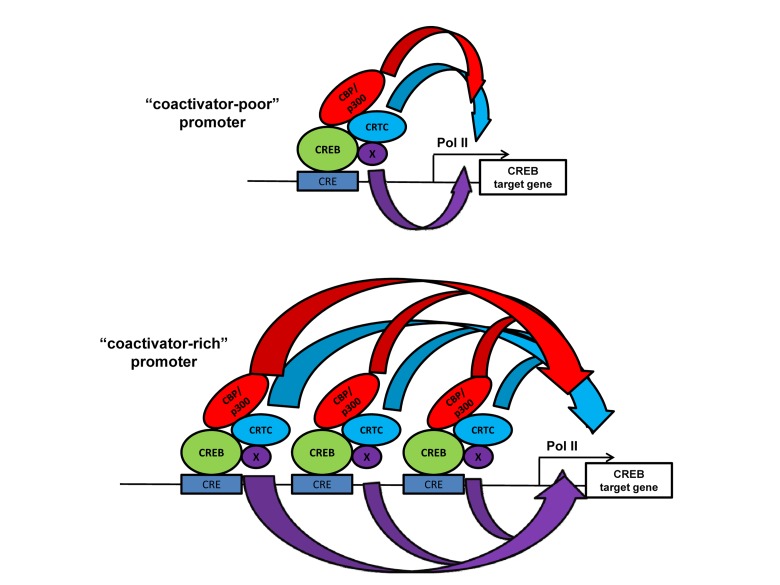
The “coactivator-poor and coactivator-rich” model showing that increased recruitment of distinct classes of coactivators (HATs CBP/p300, and non-HATs CRTC) at promoters with more bound transcription factor (CREB bound to cAMP response elements) may increase transcriptional resilience at certain endogenous target genes. Broadness of the curved arrows indicates the amount of different types (colored) of transactivating “biochemical flux” that stimulate transcription. In certain endogenous promoter contexts the increased flux though one mechanism (e.g. CRTC) may overcome the lack of a different mechanism (e.g. CBP/p300) [41]. Other types of coactivators (“x”) that might be present and participate in gene activation are shown.

Several domains of CBP and p300 (e.g. CH1 and KIX) are interconnected by peptide sequences that are not conserved and not structured (Figure 1), suggesting that such domains can function independently. Studies of CBP/p300 individual domain knock-in mutations in mice support this idea. One example is the KIX domain that binds the transcription factors CREB and Myb. CREB is a key mediator of cAMP- and calcium-inducible gene expression, and the signal-dependent phosphorylation of Ser133 of CREB is required for it to bind the KIX domain, which helps recruit CBP/p300 to target genes [63,64]. CREB also recruits the non-HAT CRTC (previously called TORC) family of coactivators in signal-dependent manner to its bZIP domain (Figure 2) [65,66]. CBP and p300 KIX domain knockin mutations alter three surface residues that interact directly with CREB and Myb [61]. Indeed, *CBP^KIX/KIX^*mice highlight the importance of this domain in CBP for learning and memory, which are CREB-mediated processes [67-69]. Analysis of *p300^KIX/KIX^* mice revealed the relevance of the domain for hematopoiesis and the specific role of the Myb interaction with p300 KIX in controlling the production of megakaryocytes and platelets [61]. Independently, a forward genetic approach corroborated these findings by identifying a different mutation on the Myb-binding surface of p300 KIX that also leads to increased platelets and megakaryocytes [70].

Interrogating cAMP-inducible gene expression in primary mouse embryonic fibroblasts (MEFs) derived from KIX domain mutant mice has refined our understanding of endogenous CREB transactivation mechanisms. Surprisingly, while some cAMP-inducible genes (which are also CREB dependent) are highly sensitive to KIX mutation, others show only partial loss of activity or are unaffected [71]. This suggests that there are KIX domain-independent mechanisms that provide compensatory coactivation functions for CREB at certain target genes. RNAi knockdown studies show that the non-HAT CREB coactivator CRTC can provide such redundancy for CBP/p300 [41]. Moreover, the overexpression of CRTC rescued the expression of some (but not all) CREB target genes that are dependent on the KIX domain or holo-CBP/p300 [41,71].

Interestingly, similar observations were observed from the study of hypoxia-responsive gene expression in MEFs deficient for both CBP and p300 CH1 domain function [72,73]. While the CH1 domain is critical for efficient recruitment of CBP/p300 to hypoxia-inducible factor (HIF) target genes and appears to contribute to an average of 35-50 percent of hypoxia-responsive gene expression, not all genes were equally sensitive to the CH1 mutation. Collectively, these findings indicate that individual endogenous CREB- and HIF-target genes differentially use multiple and partially redundant coactivator mechanisms for their expression in response to cAMP and hypoxia, respectively.

## CBP and p300 double knockout fibroblasts

Until recently, the general consensus has been that some CBP or p300 protein is required for cell viability or proliferation. For example, RNA interference (RNAi) mediated knockdown of dCBP in Drosophila Kc cells [74], and CBP and p300 in immortal HeLa cells [75], results in cell death because of chromosome shredding and mitotic catastrophe. Similarly, B and T cells lacking both CBP and p300 cannot be generated in mice, although lymphocytes that lack one or the other coactivator are viable [42-44,76]. In contrast, fibroblasts that are deficient for both CBP and p300 (double knockout or dKO MEFs) can be generated through the use of Cre/LoxP conditional knockout alleles [41]. Although dKO MEFs cannot proliferate, they are viable for weeks in culture, permitting the first assessment of transcription and histone acetylation after stable inactivation of a major family of HAT proteins.

## A non-HAT coactivator can provide context-dependent redundancy for CBP/p300

Initial experiments using CBP/p300 dKO MEFs focused on cAMP-responsive transcription. Interestingly, despite histone H4 acetylation being attenuated at CREB target gene promoters in response to cAMP, transcription was not uniformly inhibited in dKO MEFs [41]. Perhaps most surprising was the finding that the cAMP-inducible expression of several CREB target genes was actually increased in dKO MEFs. Why there are these contrasting effects of CBP/p300 loss on different cAMP-inducible genes is not clear, but there must be compensatory or redundant mechanisms that are differentially required for individual target genes [21]. Another acetyltransferase would be a logical candidate to provide redundancy, but the acetylation of certain histone residues was deficient even at target genes that were expressed strongly in the absence of CBP/p300 [41]. Instead, the non-HAT coactivators CRTC1 and CRTC2 appear to dampen the effect of losing CBP/p300 as their expression is increased in dKO MEF, and overexpression of CRTC2 can rescue the expression of certain CBP/p300-dependent CREB target genes [41,71]. Consistent with this idea, RNAi-mediated knockdown of CRTC in dKO MEFs reduces the expression of CBP/p300-independent genes, indicating that this non-HAT provides redundancy for CBP/p300 for at least some CREB targets [41].

## CBP and p300 account for nearly all H3K18 and H3K27 acetylation in fibroblasts

The effect of CBP/p300 loss on the expression of individual CREB target genes is not uniform in dKO MEFs, even though the cells have lost at least 90 percent of global histone H3K18 and H3K27 acetylation [41,77]. These mutant MEFs also have reduced promoter localized H4 hyperacetylation in response to cAMP [41]. The loss of H3K27ac is striking because this modification has been shown by others to correlate with active transcription and can be used to distinguish active enhancers from those that are inactive or poised [78-81]. Because many genes in dKO MEFs show only a partial loss of expression, or sometimes none at all, this raises questions of how, when, and where H3K18ac and H3K27ac are critical for stimulating transcription. Indeed, Valor *et al.* also noted that the loss of CBP in the forebrain neurons of adult mice dramatically reduces histone acetylation but only mildly effects transcription and cell viability [82].

## A “coactivator rich vs. coactivator poor” model for endogenous CREB targets

Curiously, increased levels of promoter-localized histone acetylation and CBP/p300 recruitment in wild type MEFs tend to inversely correlate (albeit imperfectly) with the extent to which a CREB target gene is dependent on CBP/p300 for transcription [41]. Broadly, this suggests that the importance of CBP/p300 for the transcription of a particular target gene correlates better with the low to moderate levels of recruitment of these two HATs (Figure 2). This observation has implications for interpreting genome-wide mapping studies such as ChIP-Seq, where stronger signals for a cofactor or histone modification may be interpreted to indicate functional importance.

## DNA sequence may dictate coactivator mechanisms employed by any individual gene

The critical characteristics that determine the extent to which an endogenous CREB target gene requires CBP/p300 are not completely clear, although the number of CREB binding sites may be one determining feature [41]. (This correlate does not appear to apply to plasmid reporter genes, however). Recent studies of toll-like-receptor-responsive gene expression in macrophages suggest that promoter DNA sequences that are rich in GC-content or CpG-dinucleotides might be able to overcome the need for certain types of coactivators [83,84]. However, the GC and CpG content of CREB target gene promoters does not correlate very well with their dependence on CBP/p300 for expression [41].

## Does a “coactivator rich vs. coactivator poor” model apply to other CBP/p300-dependent transcription factors and signalling pathways?

Evidence so far indicates that CBP and p300 are variably uniformly required for the function of other transcription factors besides CREB. For example, studies using dKO MEFs indicate that CBP and p300 are dispensable for transactivation of the p53 target genes, p21 and Mdm2 [85]. On the other hand, retinoic acid-inducible gene expression tends to be uniformly dependent on CBP/p300, whereas double-stranded-RNA- and serum-inducible genes show non-uniform requirements for CBP/p300 [85]. Thus it appears that the requirement for CBP/p300-mediated coactivation is context-specific for a variety of endogenous target genes driven by distinct transcription factors. Whether CBP/p300-independent target genes are generally enriched for other types of coactivators remains to be established.

## Summary

Increasingly, models of transcriptional coactivation derived from reductionist approaches do not appear to be universally applicable on a genomic scale. Data shows that CBP/p300 are recruited to many genes where they are only partly (or not all) required for stimulating transcription. A useful analogy to describe this phenomenon is to compare a DNA-binding transcription factor (e.g. CREB) to a plumber or handyman that takes a toolbox (i.e. coactivators such as CBP/p300 and CRTC) to all job sites (target genes), but it is the nature of the problem to be “fixed” that determines which tools are required at each jobsite. In this scenario, the occurrence of a cofactor at a particular locus as determined by methods such as ChIP-Seq is not indicative of function, but only suggestive. Similarly for histone modifications, loss of CBP and p300 strongly attenuates certain histone acetylation marks, notably H3K18ac and H3k27ac, but gene expression is not necessarily reduced to the same extent. Given that histone point mutations often have a modest effect in yeast, and that the effects of histone mutations in mammals are largely unknown, it seems reasonable to wonder if histone acetylation is the most important physiological function for CBP/p300.
